# Development and Validation of a Set of German Stimulus- and Target Words for an Attachment Related Semantic Priming Paradigm

**DOI:** 10.1371/journal.pone.0067684

**Published:** 2013-07-02

**Authors:** Anke Maatz, Bernhard Strauss, Karl-Jürgen Bär

**Affiliations:** 1 Institute of Psychosocial Medicine and Psychotherapy, University Hospital Jena, Jena, Germany; 2 Department of Psychiatry and Psychotherapy, University Hospital Jena, Jena, Germany; Stony Brook University, United States of America

## Abstract

Experimental research in adult attachment theory is faced with the challenge to adequately activate the adult attachment system. In view of the multitude of methods employed for this purpose so far, this paper suggests to further make use of the methodological advantages of semantic priming. In order to enable the use of such a paradigm in a German speaking context, a set of German words belonging to the semantic categories ‘interpersonal closeness’, ‘interpersonal distance’ and ‘neutral’ were identified and their semantics were validated combining production- and rating method. 164 university students answered corresponding online-questionnaires. Ratings were analysed using analysis of variance (ANOVA) and cluster analysis from which three clearly distinct groups emerged. Beyond providing validated stimulus- and target words which can be used to activate the adult attachment system in a semantic priming paradigm, the results of this study point at important links between attachment and stress which call for further investigation in the future.

## Introduction

Associations of attachment with mental illness e.g. depression [1], psychosomatic illness [2] and psychotherapeutic treatment [3]–[5] have extensively been shown and demonstrate the clinical relevance of attachment theory. This study hopes to make a contribution to research in this area by providing stimulus material that allows the activation of the adult attachment system in a semantic priming paradigm.

John Bowlby (1907–1990) developed his attachment theory drawing on the rich observations and experiences he had made in his work as a child psychiatrist [6]–[8]. It is only with Mary Ainsworth’s development of the ‘Strange Situation’ [9] however that it became possible to test his theory experimentally in the laboratory. The highly standardised, well validated paradigm allows to activate a child’s attachment system experimentally and has thus contributed to a multitude of new insights into children’s attachment behaviour and organisation [10]. When the ideas of attachment theory were expanded to adults [11], this led to a methodological drawback: until the present day, a widely accepted paradigm comparable to the ‘Strange Situation’ and allowing the experimental activation of the adult attachment system is lacking. Such a paradigm is indispensable however for assessing certain aspects of the adult attachment system, e.g. physiological and neurobiological correlates of attachment activation and organisation. A variety of paradigms have been tried, ranging from conflict resolution tasks for couples [12]–[15], the presentation of attachment themed film clips [16], [17] or the use of imaginative tasks [18]–[21] to the application of the Adult Attachment Interview AAI [22], [23] or the Adult Attachment Projective AAP [24]–[27]. Individualised stimulus material derived from the AAP has also been tried [28]. Beside having to decide whether standardised or individualised stimulus material is preferable, complexity (administration of AAI and AAP for instance require extensive training) and time intensity of administration are important to consider when choosing a paradigm. Furthermore, if physiological parameters such as heart rate, blood pressure and electrodermal activity are to be measured or if the study involves neuroimaging, experimental designs that demand the active participation of the subject are not suited.

In view of these methodological demands, semantic priming offers many attractions: It is highly standardised, once established easily applicable and it also allows for the measurement of physiological parameters. The paradigm of priming is based on the context dependence of perception and makes use of the fact that the presentation of a certain stimulus influences the reaction to a later presented target due to the organisation of memory in semantic networks [29]: semantic similarity between stimulus and target speeds up the recognition of the target. In an experimental context, subjects are first presented with a stimulus and then with a target to which they are asked to respond in a certain way. Most commonly, the target is a combination of letters and the subjects are instructed to say whether it is a word or not (lexical decision task). The presentation of the stimulus can be subliminal or supraliminal depending on whether only automatic or also controlled processes are to be assessed; the typical stimulus presentation time for subliminal priming is 20–30 ms. The priming effect is measured by calculating the difference in reaction time to the target for semantically related versus semantically unrelated/neutral primes.

Following Bowlby’s conception of inner working models [6] by which he understands unconscious cognitive structures based on internalised attachment experiences which organise perception of and reaction to current attachment-related situations, subliminal semantic priming seems well suited to assess the structure of the adult attachment system, in particular interindividual differences in the response to attachment relevant situations. Based on these theoretical considerations, Mikulincer et al. [30] devised a paradigm in which they presented subjects with either a general stress word (e.g. ‘failure’), an attachment-related stress word (e.g. ‘death’) or a neutral word and measured the reaction time to different target words which were either non-words or belonged to one of the following semantic categories: ‘neutral’, ‘interpersonal closeness’ and ‘interpersonal distance’. They found that subjects reacted significantly faster to targets associated with interpersonal closeness when primed with a stress stimulus than in the control situation with a neutral stimulus. They also found that reaction time varied significantly depending on the subject’s attachment type. From these results, it can be concluded that the paradigm activated the attachment system, an effect which has been reproduced various times since the paradigm’s first use [31]–[33]. Also in German speaking countries, some attempts to use semantic priming in the context of attachment research have been made: Banse [34] for instance used names and faces of attachment figures as stimuli, Maier et al. [35] primed their subjects with attachment-related sentences. The individualised nature of Banse’s stimuli however means that the paradigm is more work-intensive whilst being less standardised and in Maier et al.’s paradigm, the use of sentences rather than of single words creates difficulties with regards to effect attribution. A single-word priming paradigm like the one developed by Mikulincer et al. therefore appears worthwhile replicating in and for a German speaking context. This requires a set of words belonging to the semantic categories ‘emotional stress’ (general and attachment-related) and ‘neutral’ (stimulus words) along with words belonging to the semantic categories ‘interpersonal distance’, ‘interpersonal closeness’ and ‘neutral’ (target words). As German word norms have so far only been established regarding the semantic categories imageability, emotional valence [36], concreteness, valence and arousal [37], the aim of this study was to assess the semantic association of words with attachment i.e. with interpersonal closeness and distance, and thereby to provide a set of German stimulus- and target words allowing the use of semantic priming to experimentally activate the adult attachment system.

## Methods

### Ethics Statement

The study protocol was in compliance with national legislation, the principles expressed in the Declaration of Helsinki, and the Code of Ethical Principles for Medical Research Involving Human Subjects of the World Medical Association. It was approved by the local Ethics Committee (Ethikkommission am Universitätsklinikum Jena) and no research was conducted outside the authors’ institution. Participation was entirely voluntary, data were collected anonymously and could only be accessed by the first author and subjects were fully informed about the study purpose before proceeding to answer the questionnaire. As the questionnaire could reasonably be assumed not to cause subjects any harm or distress, written consent was not obtained but subjects’ decision to participate was considered to imply their consent. This procedure was in accordance with the German Society for Psychology’s research standards (Grundsätze der Forschung am Menschen, C.III, para. 6) and was equally approved by the local Ethics Committee. Psychology students were given course credits for their participation.

The study combined two methods to assess word norms, the production and the rating method [38]. In a first step, potential stimulus- and target words were identified by using the word production method. In a second step, the words sampled by the production method were rated regarding several semantic features. Both parts of the study employed online questionnaires which were administered to university students and graduates.

For the sampling of words, an online questionnaire was administered to 18 subjects who were asked to name words they associated with ‘emotional stress’ (‘emotionale Belastung’), ‘interpersonal closeness’ (‘zwischenmenschliche Nähe’) and ‘interpersonal distance’ (‘zwischenmenschliche Distanz’). The words obtained from this sampling were then assessed regarding word length (number of letters) and word frequency. These characteristics determine how difficult it is to recognise a word [39] and thus have to be matched when using words in a semantic priming paradigm. The assessment was performed using the software ‘Computergestützte Generierung und Analyse von Sprachmaterial nach Struktur- und Frequenzmerkmalen’ (‘computer supported generation and analysis of language material according to characteristics of structure and frequency’) [40]. For the target categories ‘interpersonal closeness’ and ‘interpersonal distance’, words were chosen which were between 4 and 10 letters long and which occurred with a frequency ≥10; the average word length and frequency were matched between the two categories. Stimulus words from the category ‘emotional stress’ were matched with neutral stimulus words which were added by the first author whose choice was informed by the existing sets of German affective word norms [36], [37].

23 words related to ‘interpersonal closeness’, 20 words related to ‘interpersonal distance’, 10 words related to ‘emotional stress’ and 41 neutral words were then assessed using the rating method. To this purpose, an online questionnaire was administered to 164 students who rated each word on three 7-point Likert scales regarding valence, association with emotional stress and relevance in interpersonal contexts. To ensure sufficient motivation, the questionnaire was split in half and participants were randomly allocated to answer one half or the other. It was hypothesised that semantically neutral words would yield average scores on all three scales (hypothesis 1) and that words belonging to the semantic category ‘interpersonal closeness’ would be characterised by high values for positive valence, high values for ‘relevance in interpersonal contexts’ and low values on ‘emotional stress’ (hypothesis 2). Regarding the semantic categories ‘interpersonal distance’ and ‘emotional stress’ the hypotheses were more tentative: as interpersonal distance (as e.g. expressed by the word ‘separation’), according to attachment theory, represents an emotional stressor, the question was whether the two categories would nevertheless yield distinct configurations on the three scales. It was further queried whether there would emerge two distinct categories within the category ‘emotional stress’, one for attachment-related stress and one for general stress, distinguished by their respective score on the scale ‘relevance in interpersonal contexts’. It was assumed that ‘general emotional stress’ would be characterised by low values on the valence scale corresponding to negative valence, low values on the scale ‘relevance in interpersonal contexts’ and high values on ‘emotional stress’ (hypothesis 3), whereas ‘attachment-related stress’ would be characterised by low values on the valence scale corresponding to negative valence, high values on ‘emotional stress’ but by high values on ‘relevance in interpersonal contexts’ (hypothesis 4). ‘Interpersonal distance’ was expected to be characterised by medium values regarding valence, high values regarding ‘relevance in interpersonal contexts’ and medium values on the scale ‘emotional stress’ (hypothesis 5).

The results were analysed with SPSS (version 18) using analyses of variance (ANOVA) to assess differences between the hypothesised word categories (independent variable) for each rated characteristic (dependent variable). For a more precise assessment of the categorisation, a cluster analysis was performed allowing to take the combination of the three variables’ characteristics into account. On the basis of the cluster analysis, it could then be decided for each individual word whether it belonged to the hypothesised category by assessing its distance to the cluster centre.

## Results

18 persons (13 female, 5 male, average age 26 years) answered the first questionnaire. They produced a total of 588 words and 375 different words of which 160 words were about ‘emotional stress’, 140 about ‘interpersonal closeness’ and 135 about ‘interpersonal distance’. The most frequently named words were ‘Liebe’ (‘love’) and ‘Waerme’ (‘warmth’) (11 times each) for the category ‘interpersonal closeness’, ‘Angst’ (‘fear’) (9 times) for the category ‘emotional stress’ and ‘Kaelte’ (‘cold’) (8 times) for the category ‘interpersonal distance’. Overlaps between categories were observed, especially for the categories ‘emotional stress’ and ‘interpersonal distance’.

164 individuals (133 female, 29 male, average age 22 years) answered the second questionnaire, 78 the first, 86 the second half. ANOVAS showed significant differences between the hypothesised word categories for all rated characteristics (p = 0.001). Posthoc analyses using Scheffé’s test showed that three categories are distinguished from each other by the characteristic ‘valence’, but that ‘emotional stress’ and ‘interpersonal distance’ could not be distinguished by this criterion (p = 0.561 for ‘emotional stress’ versus ‘interpersonal distance’; p = 0.001 between the three other categories). The characteristic ‘relevance in interpersonal contexts’ allowed to distinguish between words of the category ‘neutral’ and words in all other categories (p = 0.000). On the basis of the characteristic ‘emotional stress’, the category ‘interpersonal closeness’ could be distinguished from the categories ‘interpersonal distance’ (p = 0.001) and ‘emotional stress’ (p = 0.001) and the category ‘neutral’ could be distinguished from the categories ‘interpersonal distance’ (p = 0.001) and ‘emotional stress’ (p = 0.001). The categories ‘interpersonal closeness’ and ‘neutral’ and the categories ‘interpersonal distance’ and ‘emotional stress’ could not be distinguished on the basis of this characteristic (p = 0.645 and p = 0.442 respectively). These results suggested three distinct categories, ‘neutral’, ‘interpersonal closeness’ and ‘interpersonal distance and emotional stress’.

**Figure 1:**
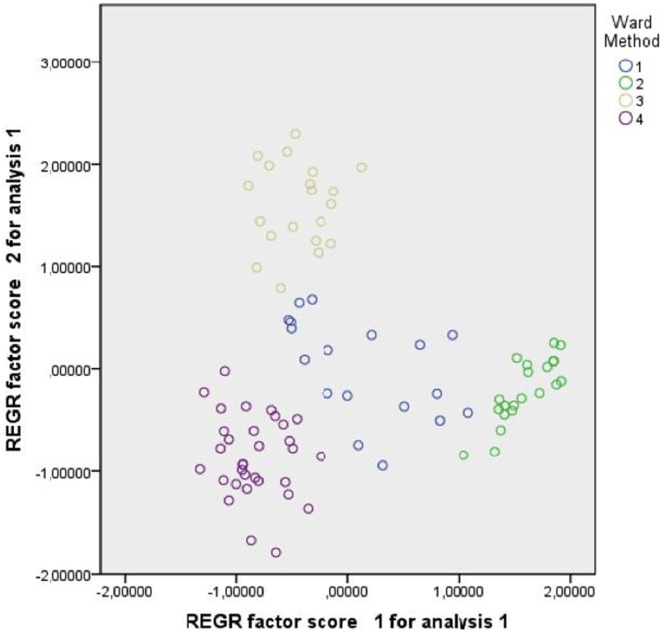
Scatterplot of the four-cluster solution. Figure 1 depicts the four clusters obtained using Ward’s hierarchical analysis with factor 1 being shown on the x-axis, factor 2 being shown on the y-axis. Cluster 1 is plotted in blue, cluster 2 in green, cluster 3 in brown and cluster 4 in purple.

The variables ‘valence’ and ‘emotional stress’ and the variables ‘relevance in interpersonal contexts’ and ‘emotional stress’ were highly correlated (r =  –0.823, p = 0.000 and r = 0.552, p = 0.000 respectively). As all variables are equally weighted in a cluster analysis and as high correlations therefore have to be prevented, a factor analysis was performed prior to the cluster analysis. This revealed two factors accounting for 67.6% (factor 1) and 30.8% (factor 2) of the variance. Taking the loading pattern of each of the former three variables into account, factor 1 can be interpreted as ‘valence’ (a negative value signifying positive valence and vice versa), factor 2 as ‘relevance in interpersonal contexts’. These factors were used for the subsequent cluster analysis which was performed following Ward’s hierarchical analysis and using squared Euclidean distances. The elbow-criterion suggested four clusters, their centres (mean values on both factors) were 0.131 (factor 1)/0.005 (factor 2) for cluster 1, 1.589 (factor 1)/−0.217 (factor 2) for cluster 2, –.0445 (factor 1)/1.601 (factor 2) for cluster 3 and –0.838 (factor 1)/−0.861 (factor 2) for cluster 4. Figure 1 depicts the four clusters with factor 1 being shown on the x-axis, factor 2 being shown on the y-axis. Cluster 1 is plotted in blue, cluster 2 in green, cluster 3 in brown and cluster 4 in purple (figure 1).

**Figure 2:**
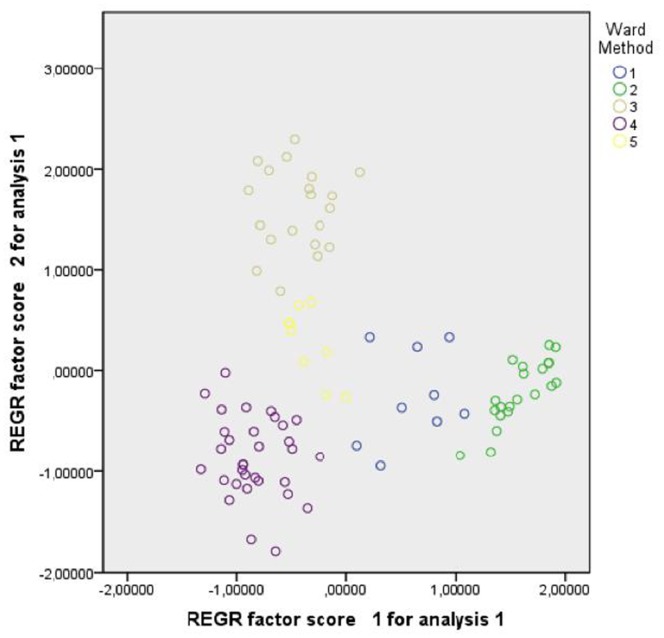
Scatterplot of the five-cluster solution. In analogy to figure 1, figure 2 depicts the clusters of the five cluster solution with cluster 1 being plotted in blue, cluster 2 in green, cluster 3 in brown, cluster 4 in purple and cluster 5 in yellow.

Comparing the solution with four clusters to the neighbouring solutions with three and five clusters showed that two clusters remained stable from the five-cluster solution on and that clusters 1 and 5 in the five-cluster solution, which in the two subsequent steps are fused with cluster 4, graphically show as a poorly defined group of points in the centre of the graph. The cluster centres in the five cluster solution were 0.604 (factor 1)/−0.261 (factor 2) for cluster 1.589 (factor 1)/−0.217 (factor 2) for cluster 2, 0.445 (factor 1)/1.601 (factor 2) for cluster 3, –0.838 (factor 1)/0.861 (factor 2) for cluster 4 and –0.341 (factor 1)/0.270 (factor 2) for cluster 5. In analogy to figure 1, figure 2 depicts those five clusters with cluster 1 being plotted in blue, cluster 2 in green, cluster 3 in brown, cluster 4 in purple and cluster 5 in yellow (figure 2).

**Figure 3:**
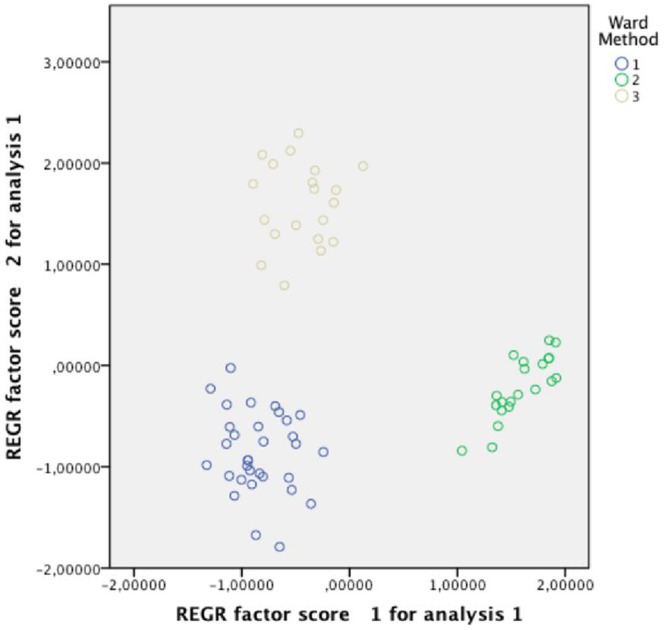
Scatterplot of the three-cluster solution after exclusion. In analogy to figures 1 and 2, figure 3 depicts the three clusters obtained after exclusion of the items/words forming clusters 1 and 5 in the five-cluster solution (see figure 2) i.e. the words which were not considered to be semantically unambiguous. Cluster 1 is plotted in blue, cluster 2 in green and cluster 3 in brown.

As it had been the aim of this study to identify words which are semantically unambiguous by assessing and thereby validating hypothesised meanings, it was decided to adopt a solution with three clusters and to exclude words belonging to clusters 1 and 5 in the five-cluster solution. In analogy to figure 1 and 2, the clusters obtained after exclusion are depicted in figure 3 with cluster 1 being plotted in blue, cluster 2 in green and cluster 3 in brown (figure 3).

These three groups consisted of 32 (cluster 1), 21 (cluster 2) and 20 (cluster 3) words respectively (the list of validated German stimulus and target words and their English translation is available as Results S1).

## Discussion

The aim of this study was to establish a validated set of words to be used as stimulus and target words in semantic priming paradigms employed to activate the adult attachment system in an experimental context. Such a set of words belonging to the semantic categories ‘interpersonal closeness’, ‘emotional stress’ and ‘neutral’ could be established.

The study was conducted by means of online-questionnaires. Despite initial reservations, this method has in recent years been shown to be a valid alternative for pen and paper questionnaires especially for sample groups who, like students, are frequent internet users and thus familiar with online and digital methods [41]. The combination of two methods to assess semantic word norms (production and rating method) alongside the combination of two methods of analysis (analysis of variance and cluster analysis) lends high validity to the results of this study.

The first two hypotheses could be confirmed: analysis of variance as well as cluster analysis revealed one group characterised by positive valence and high values on ‘relevance in interpersonal contexts’ which can be interpreted as the group of words belonging to the semantic category ‘interpersonal closeness’, and one group characterised by average valence and average values on ‘relevance in interpersonal contexts’ which can be interpreted as representing the semantic category ‘neutral’. A third group emerged characterised by negative valence along with low values on ‘relevance in interpersonal contexts’ which was interpreted as ‘interpersonal distance and emotional stress’. Neither analysis of variance nor cluster analysis allowed for further differentiation of this group and to distinguish words denoting ‘general stress’, from words associated with ‘attachment-related stress’ and ‘interpersonal distance’. The specific hypotheses 3, 4 and 5 thus had to be rejected; the tentatively hypothesised three groups in fact fall together.

The results of this study thus cannot confirm the distinction which Mikulincer et al. [30] drew between general stress stimuli (e.g. ‘failure’) and attachment-related stress stimuli (e.g. ‘death’) and furthermore shows that the stress stimuli are semantically undistinguishable from the target words of the category ‘interpersonal distance’. As Mikulincer et al. only validated their target- but not their stimulus words, this could not have been noticed. However, the group yielded different results for reaction times in the two stimulus situations: only when primed with the attachment-related stress prime did subjects with a secure attachment pattern show a reduction in reaction time to distance-related words. More recently, Nolte et al. [Brain mechanisms underlying the impact of attachment-related stress on social cognition, unpublished data] reported results from an fMRI study showing different cortical activation patterns depending on whether a general stress context or an attachment-related stress context was primed.

Regarding the present study, this raises the question whether the methods used to assess word meanings –which are indeed established and widely used methods in the assessment of semantic word norms [37] - are sensitive enough to capture subtle differences in association and connotation which do after all have an effect on our cognitive, as well as presumably emotional and behavioural response to them. When analysing the reaction times yielded in a semantic priming paradigm using the set of words established by this study, it thus has to be borne in mind that there might be subcategories within the category ‘emotional stress’. It is interesting to note however that the theoretical literature on attachment supports the results of this study rather than the distinction between interpersonal distance, general stress and attachment-related stress which is assumed in Mikulincer et al.’s as well as Nolte et al.’s studies. Bowlby conceptualised the activation of the attachment system as a response to any sort of danger, be it attachment-related like a pending separation, or not attachment-related “natural clues of danger” [6]. Mikulincer et al. name darkness and loud noises as examples of the latter category and agree that those might activate the attachment system [42] Whilst there appear to be distinct types of activators of the attachment system, it is not clear in how far the result of the activation, the state of being-activated, could be different. Viewing the results of this study against the background of the results of Mikulincer et al.’s study from which this study originated and alongside classic literature on attachment theory and recent neurobiological research on attachment theory thus raises the important theoretical question whether there is one universal way of the attachment system’s being activated or whether the result of the activation is indeed different depending on the type of stimulus. Should the latter be the case, it the specifically attachment-related nature of these processes would have to be clarified.

### Conclusion

This study makes a valuable contribution to research in attachment theory in German speaking countries by providing the material needed for the experimental activation of the adult attachment system by means of semantic priming. Moreover, the results of this study point at important links between attachment and stress which call for further investigation in the future.

## Supporting Information

Results S1 (DOCX)
