# Supplementary material for: Development and Validation of a Set of German Stimulus- and Target Words for an Attachment Related Semantic Priming Paradigm
Source: PLoS One. 2013 Jul 2;8(7):e67684. doi: 10.1371/journal.pone.0067684 (PMC3699601; doi:10.1371/journal.pone.0067684)
Supplement: Results S1 — (DOCX) [file pone.0067684.s001.docx]

Validated German stimulus and target words (and their English translation)

Nähe (interpersonal closeness)

Sicherheit (security)

Liebe (love)

Mutter (mother)

Wärme (warmth)

Hilfe (help)

Beziehung (relationship)

Kontakt (contact)

Offenheit (openness)

Gelassen (relaxed)

Nähe (closeness)

Vater (father)

Freund (friend)

Vertraut (familiar)

Trost (consolation)

Herzlich (cordial)

Respekt (respect)

Zärtlich (tender)

Lächeln (smile)

Sprache (language)

Schützen (protect)

Distanz (interpersonal distance)

Niederlage (failure)

Krankheit (illness)

Schmerz (pain)

Tod (death)

Trennung (separation)

Angst (fear)

Streit (argument)

Ärger (anger)

Hass (hatred)

Lüge (lie)

Misstrauen (mistrust)

Vorwurf (reproach)

Krank (ill)

Verlust (loss)

Verlassen (abandoned)

Gewalt (violence)

Ablehnung (rejection)

Enttäuscht (disappointed)

Verletzung (hurt)

Aggression (aggression)

Drohung (threat)

neutral (neutral)

Flugzeug (airplane)

Maschine (machine)

Fabrik (factory)

Stuhl (chair)

Kiste (box)

Fahrrad (bike)

Landschaft (landscape)

Zeitung (newspaper)

Boot (boat)

Papier (paper)

Sessel (armchair)

Koffer (suitacse)

Sendung (broadcast)

Sammlung (collection)

Treppe (stairs)

Trinken (drink)

Fenster (window)

Schrank (wardrobe/cupboard)

Wasser (water)

Balkon (balcony)

Radio (radio)

Fahren (go)

Tasche (bag)

Heizung (heating/radiator)

Schiff (ship)

Bibliothek (library)

Autobahn (motorway)

Technik (technology)

Schlüssel (key)

Tür (door)

Rechnen (calculate)

Wetter (weather)
